# A Law of Redundancy Compounds the Problem of Cancer and Precision Medicine

**DOI:** 10.1007/s00239-023-10131-2

**Published:** 2023-09-04

**Authors:** Rama S. Singh

**Affiliations:** Professor Emeritus, Department of Biology and Origins Institute, McMaster University, 1280 Main Street W., Hamilton, ON L8S 4K1 Canada

**Keywords:** Mutation, Complexity, Redundancy, Hidden variation, Precision medicine, Cancer

## Abstract

Genetics and molecular biology research have progressed for over a century; however, no laws of biology resembling those of physics have been identified, despite the expectations of some physicists. It may be that it is not the properties of matter alone but *evolved properties of matter* in combination with atomic physics and chemistry that gave rise to the origin and complexity of life. It is proposed that any law of biology must also be a product of evolution that co-evolved with the origin and progression of life. It was suggested that molecular complexity and redundancy exponentially increase over time and have the following relationship: DNA sequence complexity (Cd) < molecular complexity (Cm) < phenotypic complexity (Cp). This study presents a law of redundancy, which together with the law of complexity, is proposed as an *evolutionary law of biology*. Molecular complexity and redundancy are inseparable aspects of biochemical pathways, and molecular redundancy provides the first line of defense against environmental challenges, including those of deleterious mutations. Redundancy can create problems for precision medicine because in addition to the issues arising from the involvement of multiple genes, redundancy arising from alternate pathways between genotypes and phenotypes can complicate gene detection for complex diseases and mental disorders. This study uses cancer as an example to show how cellular complexity, molecular redundancy, and hidden variation affect the ability of cancer cells to evolve and evade detection and elimination. Characterization of alternate biochemical pathways or “escape routes” can provide a step in the fight against cancer.

## Introduction

The existence of life appears to be a paradox when considering the composition of the inanimate universe. Life not only originated in the inanimate universe, but also evolved and became successful through the creation of a wide variety of organisms that now inhabit or once inhabited the Earth (Darwin 1859; Mayr 1963; Gould 2002; Carroll 2005). The evolution of living things is no longer considered a violation of the Second Law of Thermodynamics, as that of a closed, energy-consuming system of increasing complexity (Lewontin 1968). Origin of life is an active field of research (Malaterre et al. 2022; Preiner et al. 2020) and how to define life (Benner 2010) has taken on an added importance due to its relevance to space exploration; however, the unanswered paradox that remains is this: “What differentiates the living from the nonliving?” or, in the language of physicists, “What properties of matter or physical laws gave rise to the origin and evolution of life forms?”. Bohr (1932, cited in Bohr 2010) introduced the principle of complementarity, which states that paradoxical observations such as that of light presenting both particle and wave properties can be explained through comparable results of independent experiments. Bohr proposed that the paradoxical appearance of animated beings from inanimate sources may be similarly explained by the complementarity principle of biology (Bohr 1932). This proposition became the basis of the work on virus replication by Max Delbrück, which was undertaken to understand complementarity in biology (Strauss 2017).

In the early twentieth century, there was a belief among physicists that new laws of physics would be found in the field of biology. Delbruck (1949) reasoned that these laws may not be unique to this field and hypothesized that as “the processes of living matter must be essentially the same as those of the inorganic world and that *there could not possibly exist a biological science ruled by its own laws* [emphasis added]” (Delbruck 1949, p. 5/9)*.*

However, Delbrück subsequently altered this hypothesis and became convinced of the enormity of the cellular complexity and stated that: “the meanest little cell becomes a magic puzzle box full of elaborate and changing molecules, and far outstrips all chemical laboratories of man in the skill of organic synthesis performed with ease, expedition, and good judgement of balance.…*any living cell carries with it the experiences of a billion years of experimentation by its ancestors* [emphasis added]” (Delbrück 1949, p. 2).

Recently, a hypothesis of complexity was presented to clarify the underlying mechanisms of complex traits including those of complex diseases (Singh and Gupta 2020), whereby molecular complexity and redundancy were suggested to increase exponentially over time. Complexity implies a network of gene–gene interactions, redundancy, a flow of information through the network of biochemical pathways. Three types of complexity were suggested with the following relationship: Phenotypic complexity (Cp) > molecular complexity (Cm) > DNA sequence complexity (Cd) (Singh 2021). DNA complexity is defined here as DNA sequence complexity, molecular complexity as complexity in all cellular molecules, and phenotypic complexity as all aspects of structural phenotypic complexity above the molecules. This inequality is not simply quantitative but it implies “many to fewer” relationships in the flow of information content from DNA to proteins, and from proteins to phenotypes. The concept of redundancy was elaborated through the idea of *hidden variation* and the proposition that two kinds of variation exist: *segregating variation* in genome sequences *and non-segregating or fixed regulatory variation* in biochemical pathways (Singh 2021). Organisms were assumed to use both of these methods of variation for adaptation, with segregating variation more readily adopted than hidden variation.

In this article, we extend the discussion of redundancy and consider its implications for precision medicine with special reference to cancer (Hanahan 2014; Huang 2014; Lichtenstein 2019; Sonnenschein and Soto 2020). A law of redundancy is presented, and it is argued that the interconnecting network of biochemical pathways allows redundancy (alternate pathways) to evolve, thereby intertwining complexity and redundancy in an interdependent and inseparable manner in the origin, evolution, and complexity of life. Molecular redundancy in biochemical pathways would be the first line of defense against environmental challenges and the harmful effects of deleterious mutations. The law of redundancy applies to all aspects of biology, from serving as an additional, hidden source of evolutionary variation to the advancement of complex diseases. Complexity can create problems for precision medicine because of the involvement of a multitude of genes, while redundancy implying hidden variation and the existence of alternate pathways among genotypes and phenotypes could challenge the detection of genes related to complex diseases and mental disorders. Variation and evolution of cancer is a major problem of our time and this study uses cancer as an illustrative example to show how hidden variation and molecular redundancy could allow cancer cells to evolve and evade detection and elimination.

## Origin and Importance of Molecular Redundancy

Details of the mechanisms for the origin of redundancy are presented elsewhere (Singh 2021). Ever since the publication of Ohno’s “Evolution by Gene Duplication” (Ohno 1970) redundancy is generally equated with “extra, unnecessary, and spendable” information. For example, a duplicated copy of a gene is seen as extra and unnecessary for the particular function, although gene duplication provides material not only for the evolution of new genes but it also resets the dosage relationships and gene expression of the entire genome. It is important to point out here that by redundancy here we mean redundancy in information arising from alternate biochemical pathways controlled by gene networks. In the following we show why redundancy is important and why and how it may have been favored in the evolution of both single cell and multicellular organisms (Kozolov 2014).

In 1950, before the discovery of DNA, H. J. Muller wrote a seminal paper titled “Our Load of Mutations,” in which he discussed the risks of high rates of mutation caused by ionizing radiation and the accompanying threats to human populations (Muller 1950). He stated that high rates of deleterious mutations and their negative effect on fitness, in combination with declining rates of reproduction, threatened human health and could lead to our extinction. Muller suggested that a method of artificial selection should be imposed, such as voluntary abstention from reproduction to prevent the elimination of approximately 20% of the global population due to genetic inadequacy.

Muller was referring to mildly deleterious mutations that could endure for a long period and possibly fix themselves in small populations due to random drift. Sexual reproduction has the ability to eliminate deleterious mutations from populations, because sexual organisms carry varying numbers of these mutations, and those with the highest number are more likely to be selectively removed from the population due to death or the absence of reproduction (Muller 1947; Crow 2000; Human Genome Project Consortium 2012). In addition, sexual reproduction could provide a second mechanism to reduce the number of deleterious mutations through an increased fidelity of DNA replication; however, because of the increasing amount of DNA in the cell, the number of deleterious mutations carried by a sexually reproducing individual would still be high. The 1000 Genome Project consortium showed a mutation rate in the range of 1.0 × 10^–8^ to 1.4 × 10^–8^, with an average of *µ* = 1.1 × 10^–8^ per nucleotide (HGP 2012). Similar values were obtained based on sequencing, and a newborn baby would carry an average of 100 de novo mutations (Lynch 2016). Assuming that most deleterious mutations have a fitness loss of approximately 1% and that they embody 1–10% of all mutations, the mutation rate for these mutations would be 1 per diploid genome, with a 1% fitness loss per generation (Lynch 2016). This is a heavy mutation load.

Life is 3.5 billion years old, and sexual reproduction is estimated to have evolved 2 billion years ago, beginning with the exchange of genetic material between prokaryotes; therefore, life was *asexual* for the first 1.5 billion years (i.e., for 40% of the time life existed on this planet). How did organisms deal with deleterious mutations prior to the existence of sexual reproduction? We can imagine the enormity of the problem based on somatic mutation rates in complex organisms. In all species for which data are available, somatic mutation rates are substantially higher than germline rates (Lynch 2009, 2010). While tissues vary in somatic mutation rates (Tomasetti et al 2013; Lodato et al 2015; Martincorena et al 2015), the average rate is approximately 50 times higher than that of germline mutations (Lynch 2016). Humans have 10^13^ cells in their bodies, which means that an adult would carry approximately 10^16^ mutations, with every nucleotide site having mutated in thousands of cells. Although at the beginning of life, when there was less DNA, the total number of mutations in asexual cells would be smaller, there were no mechanisms for purging deleterious mutations except by selection. The rate of mutation would have increased as a function of the amount of DNA in the cell, which was tempered by a decrease due to improvements in DNA replication fidelity.

The problem of mutation is not that the rate of mutation would be high, but that (1) at any rate of mutation and at any time in the history of evolution, the proportion of deleterious mutations would be far greater than the proportion of beneficial mutations, and (2) the probability of deleterious mutation effects would increase as a function of the increasing number of gene–gene interactions due to the greater number of genes and proteins in complex organisms (Yang et al 2003). Therefore, mutation is both a *constructive force,* as it is the ultimate source of variation for evolution, and a *destructive force,* as it presents a challenge to organisms, which build gene complexity through selection and evolution. Asexual organisms could only mediate the effects of deleterious mutations through beneficial mutations and genetic variation, and these processes would be rare in the absence of sexual reproduction and recombination. Molecular redundancy would have given them recourse from this situation. The redundancy would have evolved from a single-cell organism with gene–gene interactions, whereby one gene affected many functions, redundant pathways, and physiological redundancies (Noble and Hunter 2020; Singh 2021). Redundancy would have expanded in sexual organisms with gene duplication, copy number variation, increased metabolic pathways, cell-specific variants, non-coding DNA, and other factors. Bacteria have a streamlined genome with few duplicated genes and little non-coding DNA, but this does not indicate that redundancy did not evolve in their biochemical pathways. Redundancy is treated here as a system that protects organisms from harmful mutations but does not necessarily lead to improved adaptation (Frank 2007; Lynch 2012).

## A Law of Redundancy

Previous studies have focused on complexity and redundancy in terms of their origin and evolution, and complex trait variation effects, including those relating to complex diseases (Singh and Gupta 2020; Singh 2021). Here, we generalized the role of redundancy in the origin of life and complexity discuss its implication for evolution in general and cancer in particular. A law of redundancy can be constructed based on the following set of premises, which are derived from the relationships between mutation and complexity as described above.

1. Mutations, regardless of time, place, or rate, result in deleterious mutations considerably more often than they result in non-deleterious mutations.
2. The probability of deleterious mutations would increase with increasing protein subunit complexity, gene–gene interaction, and single proteins affecting numerous functions.
3. Biochemical pathways are extended by the augmentation of existing pathways; therefore, old routes would provide access to new routes and alternate/redundant pathways would enable new pathways, thus increasing complexity and greater numbers of genotype–phenotype relationships.
4. The initial redundancy arising from the evolution of network complexity would have been subsequently augmented by DNA structural-expression redundancy arising from gene duplication and molecular and historical contingencies.
5. Redundancy provides a mechanism, besides gene-environment interaction, for the norm of reaction.
6. Molecular redundancy provides cells with the capacity to compensate, even if partially, for mildly deleterious mutations, thus allowing for the evolution of increased functional complexity.
7. Molecular redundancy provides the organisms with the first line of defense against short-term fluctuations in environmental conditions through phenotypic plasticity and/or behavioral self-protection.
8. Molecular redundancy, beside horizontal gene transfer (Emamalipour et al 2020) and new beneficial mutations (Desai et al 2007), would have been the only mechanism to modulate the effects of deleterious mutations for the first 1.5 billion years of (asexual) evolution.

A law of redundancy can be stated as follows:

The evolution of gene networks has provided cells with additional methods to evolve through alternate, redundant biochemical pathways, thereby enabling cells and organisms to present a first line of defense against environmental challenges, including mildly deleterious mutations, to allow molecular complexity to progress.

The relationship between cellular complexity and redundancy can be exemplified by the road network of an old city. Similar to intersecting networks of streets that evolve to serve a complex city, intersecting networks of biochemical pathways evolve to serve complex cells and the body. The flow of traffic in the city is regulated efficiently by minor re-routing in the short term and major re-routing and new road construction in the long term, and networks of biochemical pathways ebb and flow through the use of physiological redundancy (Noble and Hunter 2020) in the short term and by increasing in complexity with layers of new pathways in the long term (Singh 2021). Similarly, as existing old roads provide access for building new roads, existing biochemical pathways and gene networks enable the addition of new nodes in the network of cellular complexity. Molecular complexity and redundancy are inseparable aspects of the same phenomenon. The companion laws of complexity and redundancy would fit the criteria of Shrodinger’s “new laws to be expected in the organism” (Schrondiger 1944).

## Redundancy and Hierarchical Genome Complexity

Over the last hundred years, Mendelian genetics and molecular biology have cemented the gene centric view of evolution. Evolutionary geneticists have always held that the overall fitness of a gene must depend on the rest of the genes in the genome, and currently there is an active interest in systems biology (Trewavas 2006; Barbieri 2018; Dhillon et al 2020), genome architecture (Koonin 2009; Lynch et al 2006; Flaxman et al 2014), and karyotype coding (Heng and Heng 2021; Heng et al 2023). The evolution of organisms and their molecular and phenotypic complexities are finally beginning to be seen as the result of a multilevel process of gene regulation and fitness modification by adaptive and non-adaptive processes working together but at different time scale.

In our treatment of complexity we have focused at three levels: DNA sequence complexity (Cd), molecular complexity (Cm), and phenotypic complexity (Cp) with the relationship as: Cd < Cm < Cp (Singh and Gupta 2020). The organization of DNA sequences in the genome is heterogeneous due to a variety of processes and these include gene duplication, deletion, copy number variation, transposition, inversion, non-coding DNA, recombination, different chromosomal element, translocation, and polyploidy. Since the DNA sequence elements affected by these processes would vary in their rates and evolutionary dynamics, the levels of complexity will also vary for each type of these DNA elements. In view of the discussion on systems biology and genome architecture, we can define three levels of DNA complexity and with the following relationship: karyotype complexity (Ck) < genome architecture complexity (Ca) < DNA sequence complexity (Cd). The full range of the relationships of the genomic and phenotypic complexities can be represented as follows: Ck < Ca < **Cd** < Cm < Cp. The inequality signs among the various types of DNA complexity reflect inequality in the number of respective mutational elements and the amount of standing genetic variation. The inequality signs above DNA sequence complexity reflect inequality in the amount of information. Genetic elements that do not produce standing variation in populations cannot be the source of adaptive evolution and their fate in populations may largely be governed by historical processes. It is not surprising that while the “functional genome” (based on phenotypic variation) is largely the basis of organismal evolution by natural selection, the structural genome (sequence evolution) appears to be largely shaped by non-adaptive processes (drift and mutation).

It would not be farfetched to say that the whole field of genomics especially its role in precision medicine is presently going through what the Atomic Physicist Niels Bohr called “chaos” (Bohr 1961). Bohr remarked that “development in a new field will usually pass through stages in which chaos becomes gradually replaced by order” (Bohr 2010, p. 66). The concept of redundancy may help reduce this chaos.

## Redundancy and Hidden Variation: The Norm of Reaction and Plasticity

From an evolutionary perspective, organisms and populations have two conflicting requirements: retain ontogenetic stability of the body form over generations and respond to evolutionary pressures between generations. The dynamics of this stability within generations and change between generations is the crux of the contrasting views between developmental biologists and evolutionists on modern synthesis (Mayr 1963; Gould 2002; Carroll 2005; Noble 2021). Because the nature of *genetic* vs. *developmental* variations may impact the speed of evolution, their respective roles in stability vs. change and microevolution vs. macroevolution has been long debated in evolutionary biology (see Noble 2021 and references therein).

Redundancy is an essential factor, along with mutation and recombination that affects variation; however, it also affects both the nature of (hidden) variation and the dynamics of selection through its effect on genotype and phenotype relationships. By hidden variation we mean, variation represented in the form of alternate pathways and of course invisible in DNA sequences. Redundancy provides pathway options from genotypes to phenotypes, thereby allowing genotypes and organisms to negotiate their trajectories on the fitness landscape (Wright 1988; Gonzalez-Forero 2023). As an example, we use the concept of a reaction norm to show the resultant phenotypic plasticity, from genotypic responses in varying environments.

**Fig. 1:**
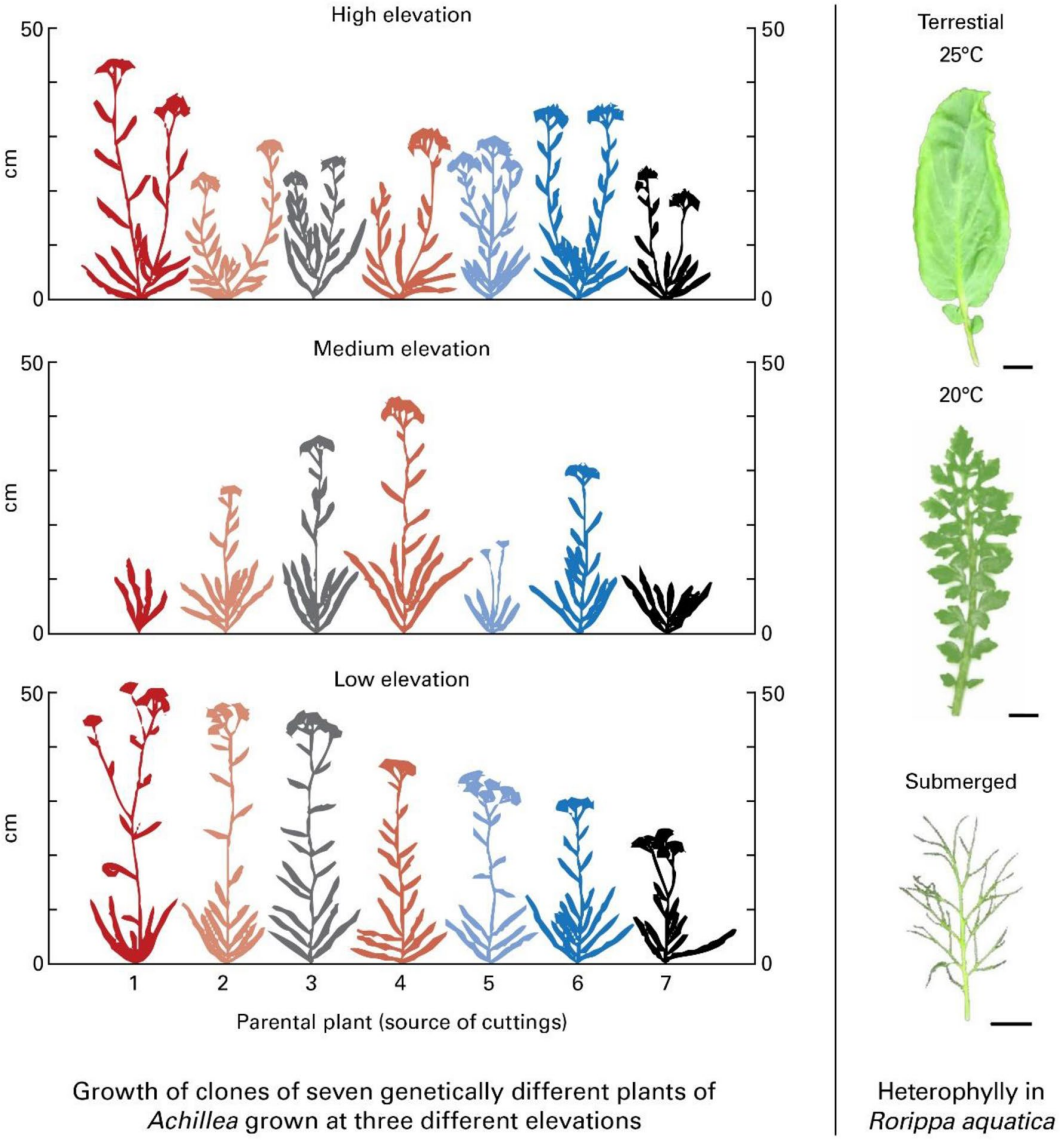
Examples of quantitative (left panel) and qualitative (right panel) norms of reaction. Left panel: *Achillea millefolium*: Norm of reaction to elevation for seven different *Achillea* plants (seven different genotypes). A cutting from each plant was grown at low, medium, and high elevations (Carnegie Institutions of Washington/ from A. J. F. Griffiths et al. 1996. An Introduction to Genetic Analysis, p. 16, Sixth Edition. Freeman, permission of the Author). Right panel: *Rorippa aquatica*: Heterophylly in *Rorippa* plants showing three distinct types of leaf in three different environments varying in temperature and underwater condition (From Li et al. 2019)

The two types of variation proposed above (genetic and developmental) can be related to a norm of reaction through the two examples presented in Fig. 1. The classic genotype x environment interaction from the *Achillea* research of Clausen et al. (1948) is an example of the role of genetic variation and a *quantitative* response (Fig. 1 left panel). The case of heterophylly in the North American lake cress, *Rorippa aquatica* (Fig. 1, right panel), is an example of the role of developmental variation and a discrete, *qualitative* response. A systematist who is unaware of heterophylly may classify the three forms shown in Fig. 1 (right panel) as three separate species, each adapted to their own niche. A norm of reaction may not be simply the outcome of a genotype × environment interaction but could involve variation hidden in alternate pathways of the traits. The distinct developmental forms of lake cress that were produced by the same genotype in distinct environments parallel the effects of a sustained selection pressure over time in terms of speciation and macroevolution using hidden variations. Disregarding the role of major genes affecting qualitative traits, redundancy combined with a norm of reaction overrides arguments based on genetic determinism for complex traits, including complex diseases and behaviors.

## Redundancy and the Problem of Variation in Evolution

Elucidation of the structural—functional basis of variation has been the driving force of evolutionary biology. Mendelian genetics solved Darwin’s problem of variation and provided a basis for the maintenance of genetic variation, and together with the selection and mutation gave rise to the problem of genetic load (Wallace 1987) and mutation load (Muller 1950). Molecular investigations of genetic variation discovered that there was too much variation and introduced the concepts of “neutral” molecular variation and evolution (Lewontin 1974; Kimura 1983). For nearly a hundred years, single gene dynamics dominated population genetics. The Genomics revolution turned things around. Genome sequencing and precision medicine studies showed that there were not enough genes to code for different traits uniquely, and that complex traits were controlled by many genes of small effects, and introduced the concepts of “missing heritability,” gene network, and pervasive gene interaction (see Singh 2021 for a recent review).

Genomics has revolutionized developmental biology and studies of the relationship between genotypes and phenotypes. Molecular redundancy helps clarify the relationships between genotypes and phenotypes and enables developmental biologists to ask questions such as this: considering that there are an insufficient number of genes to form all traits separately and genes are shared between traits, and since epistasis is universal features of the functional genome, what determines developmental boundaries, rates, or durations of trait-specific genetic information translations during ontogeny? Along with hidden variation and alternate pathways (Singh and Gupta 2020; Singh 2021), we must consider not *dedicated* but *distributed genotypes* in relation to complex traits*.* Genes code for enzymes and influence traits through enzyme-controlled biochemical pathways, so just as genes are the basis of all living forms, so is redundancy which is simply the result of the multiplicity of the biochemical pathways.

Redundancy applies to all life forms and is relevant to many problems of biology—some old, some new. First, molecular redundancy extends the scope of gene-environment interaction and norm of reaction (Lewontin 2000), softens the pressure of mutation load (Muller 1950), enables evolution of alternate adaptive peaks (Wright 1988), and provides hidden variation for macroevolution (Goldschmidt 1940; Gould and Eldredge 1977). It would have also facilitated initial conditions for the origin of life (Malaterre et al. 2022).

Second, redundancy can provide new perspectives on genetic variation and we will mention three: (1) Genome sequencing studies (Ponting and Hardison 2011) and the ENCODE Project Consortium (2012) grossly vary in their estimates (~ 15% vs. 80%, respectively) of what proportion of the human genome is functional. Redundancy may not help to determine what proportion of the human genome is functional (Gaur 2017; Galeota-Sprung et al 2020) but it can explain why and how even a relatively small proportion of the functional genome can go farther through gene interaction network and multiplication of pathways; (2) Redundancy provides an alternate perspective on the concept of “neutral variation” (Kimura 1983). Under redundancy the fitness of a gene at a given time and place is not a reflection of its true potential in varying conditions. Redundancy can help build new theories of “constructive neutralism” and explore conditions under which neutral or nearly neutral genes may become evolutionarily useful (Wilke 2001; Massel et al. 2007; Lynch 2012); and (3) Redundancy can provide mechanism for what has been called “developmental system drift” to explain the stability of phenotype(s) in the face of genetic divergence between species (Gruber et al 2016; Cauret et al. 2020; Ewe et al 2020), as well as for the idea that a given polypeptide may not necessarily assume a native shape dictated by the gene sequence (Baverstock 2019) and proteins can perform more than one role in an organism (Gancedo et al 2016).

Redundancy is relevant to the problem of ontogeny and origin of morphological innovations and evolutionary novelties but this is outside the scope of this article.

Finally, redundancy provides a molecular basis for the biological basis of human freedom (Dobzhansky 1956) and the evolution of choice and an explanation for Kant’s biological teleology (Roll-Hansen 2000; McKaughan 2005).

In the following, because of their importance to human health, we have selected precision medicine and cancer as illustrative examples to show how redundancy may modulate the relationship between genotypes and phenotypes and provide escape routes to cancer cells.

## Redundancy is Problematic for Precision Medicine

Science promises more than it can deliver, at least in the short run. This is also true of precision medicine. The big promises of the Human Genome Project were not fulfilled because complex diseases and mental disorders are affected by many genes, most of which produce small effects, which is not ideal for precision medicine (see Singh 2021 and references cited therein). Next generation sequencing in combination with big data, bioinformatics, and AI promises to open new frontiers in personalized medicine (Denny and Collins 2021; Morash et al 2018; Xu et al 2019). Precision medicine has so far focused on biological determinants and it has been argued that a more personalized approach using environmental, socio-economic, and psychological factors in addition to biological determinants, would be more productive (Delpierre and Lefevre 2023).

Molecular redundancy compounds the problem of precision medicine in two ways: First, it creates alternate pathways between genotypes and phenotypes. Alternate pathways not only create “many to many” relationships between genes and traits, but they also end up making the effects of individual genes necessarily small. The combined effect of complexity and redundancy is that two individuals sharing the same risk factors may not show the same disease. This has been discussed in detail elsewhere (Singh 2021). A second way, we show below, using cancer as an example, how molecular redundancy and hidden variation may allow cancer cells to evolve and evade detection and elimination.

## Origin, Evolution, and Persistence of Cancer Cells

Cancers behave as evolving entities, yet no unifying theory exists regarding the origin and proliferation of cancer (see Sonnenschein and Soto 2020 and references cited there in). The mainstream theory postulates that cancers are caused and driven by somatic mutations (Knudson Jr. 1971; Bast Jr. et al. 2009; Papaemmanuil 2013; Martincorena et al. 2015). For example, mutations in the gene Tp53 are involved in nearly 50% of human cancers, and these mutations affect tumor suppression (Wang et al 2012; Muller and Vousden 2013; Hientz et al 2016). Successive mutations (e.g., in oncogenes, growth factor signals, and DNA repair genes) increase rapid clonal cell proliferation and provide these cells with fitness advantages over non-proliferating normal cells, leading to tumor formation and metastasis (Nowell 2013; Greaves 2018). As the prevalence of somatic mutations increases with age, significant health problems occur (Jin 2010; Manders and Middelkamp 2021). In mice and humans, somatic mutations are almost two orders of magnitude higher than germline mutation (Milholland et al 2017).

How cancer cells originate is not entirely clear. For Breast Cancer, for example, two models have been proposed: Sporadic mutation model and the stem cell model (see Bombonati and Sgroi 2011 and references cited in there). Under the sporadic mutation model, any epithelial cell with random mutations and cell proliferation can lead to tumor development. In the stem cell model, only a small subset of tumor cells, stem and progenitor cells, will lead to tumor development. Regardless of which model is correct (Bombonati and Sgroi 2011), the theory of redundancy provides a new framework for the origin, evolution, and persistence of cancer cells.

It is proposed that cancer cells behave like semi-autonomous entities with the capacity “to fight” for self-protection, evade threats, change, and evolve. Cancer cells do this using physiological redundancy in biochemical pathways in addition to new mutations (Singh 2021). Given the pressure of somatic deleterious mutations, somatic cells can escape death by using alternate pathways and need not wait for new advantageous mutations. Population genetics experiments suggest that the release of hidden variation and response to selection and evolution of robust pathways are expected to be slow (Mather 1943; Waddington 1953; Rendel 1959), however under strong and persistent selection pressure which cancer drugs provide, progress in clonal evolution is imminent.

Many common observations regarding cancers can be explained by redundancy and hidden variations, including the initial slow growth of cancer cells that is followed by rapid proliferation, genetic heterogeneity, variation in gene expression, resistance to drugs, immuno-evasion, dormancy, and resurgence (Geiger and Peeper 2009; Hanahan and Weinberg 2011; Bombonati and Sgroi 2011; Shortt and Johnstone 2012; Bennett et al 2022; Guo et al 2023). Under the theory of redundancy, genetic markers may not be a true reflection of tumor’s physiological potential to change and evolve using hidden variation in alternate pathways as reflected by the chaotic nature of bioprocesses during health crises.

## Conclusion

The sequencing of the human genome was expected to result in advanced, genome-based precision medicine, but this has not yet materialized. As genes for complex diseases and mental disorders were identified and mapped, it became clear that these diseases were not generally caused by major genes, but by various genes with small effects and many of these genes affected other diseases. To provide an evolutionary framework for personalized medicine, we presented a theory of complexity and introduced the concepts of unnecessary complexity and redundancy and *hidden variation* in biochemical pathways, which contrasted with the *segregating variation* in the genome (Singh and Gupta 2020; Singh 2021).

In this study, we extended the origin of redundancy back to the origin of life as a mirror image and companion of complexity. Complexity implies a network of gene–gene interactions, redundancy a flow of information through the network of biochemical pathways. Physiological redundancy would have provided the first line of defense to organisms against environmental challenges including deleterious mutations, especially during the first 1.5 billion years of asexual evolution. Along with mutation and recombination, redundancy is a major determinant of molecular variation for evolution.

The concept of redundancy provides a window to the relationship between genotypes and phenotypes of complex traits as well as to the long-term developmental dynamics of organisms and their genomes. Complexity and redundancy pose problems for precision medicine because, in addition to the problem arising from the involvement of a multitude of genes, redundancy implies hidden variation and the existence of alternate pathways among genotypes and phenotypes, which could create challenges in detecting genes for complex diseases and mental disorders. We believe that some of the cellular and molecular behaviors of cancer cells cannot be understood without implicating hidden variation and redundancy that provide these cells with a partial level of cellular autonomy. Characterization of alternate biochemical pathways or “escape routes” would be the next step in the fight against cancer.
